# *Helicosphaera carteri* (Prymnesiophyceae) under high carbon dioxide: An experimental study

**DOI:** 10.1111/jpy.70103

**Published:** 2025-11-24

**Authors:** Stefania Bianco, Manuela Bordiga, Gerald Langer, Patrizia Ziveri, Federica Cerino, Federica Relitti, Vincenzo Alessandro Laudicella, Andrea Di Giulio, Claudia Lupi

**Affiliations:** ^1^ Department of Earth and Environmental Sciences University of Pavia Pavia Italy; ^2^ National Institute of Oceanography and Applied Geophysics – OGS Trieste Italy; ^3^ Institute of Environmental Science and Technology (ICTA), Universitat Autònoma de Barcelona (UAB) Bellaterra Spain; ^4^ Catalan Institution for Research and Advanced Studies (ICREA) Barcelona Spain; ^5^ Department of Animal Biology Plant Biology and Ecology (BABVE) Universitat Autònoma de Barcelona Bellaterra Spain

**Keywords:** coccolithophores, *Helicosphaera carteri*, ocean acidification

## Abstract

The coccolithophore *Helicosphaera carteri* is an understudied, yet ecologically and biogeochemically important, marine calcifier. Hence, its response to ocean acidification has implications for ecosystem function and the marine carbon cycle. Here we employed dilute batch cultures featuring a coupled C‐system manipulation (295, 444, and 600 μatm CO_2_) to analyze the response of *H. carteri* in terms of growth rate and particulate carbon production, two key eco‐physiological and biogeochemical parameters. We highlight that both growth rate and organic carbon production are CO_2_ limited at 295 µatm but are not proton inhibited at 600 µatm of CO_2_. This finding, combined with the maintenance of a stable inorganic production rate, places *H. carteri* among the coccolithophores less sensitive to seawater acidification. In addition, we tested a widely applied assumption underpinning the determination of carbon production, namely the constancy of particulate carbon quotas over the course of a dilute batch culture. We determined that the assumption holds true, an important validation of a method used in many publications.

**Abbreviations**
∅: coccosphere size
μ: growth rate
Θ: protoplast size
AR: aspect ratio
CaCO_3_
: calcium carbonate
CO_2_
: carbon dioxide
CRM: certified reference material
C‐system: carbon system
*d*
: short axis cell diameter
DIC: dissolved inorganic carbon
IPCC: Intergovernmental Panel On Climate Change
OA: ocean acidification
OGS: National Institute of Oceanography and Applied Geophysics
PBR: photobioreactor
PIC: particulate inorganic carbon
POC: particulate organic carbon
RD: roundness
SEM: scanning electron microscopy
SOP: standard operating procedure
SSP: Shared Socioeconomic Pathways
TA: total alkalinity
*V*_cell_
: protoplast volume

## INTRODUCTION

Interest in understanding the effects of ocean acidification (OA) on coccolithophores has led to the investigation of responses of these organisms to changes in seawater carbon chemistry not only across different species and strains but also across different aspects of these organisms, such as morphology, morphometry, calcification, and photosynthesis rates (e.g., Bach et al., 2015; Fiorini et al., 2011; Langer et al., 2011; Langer & Bode, 2011). However, all the studies conducted so far have highlighted the species‐specificity of coccolithophores' responses to OA and, consequently, the need to conduct more experiments, testing new species and strains. Another important aspect to consider is the fact that carbon production in typical coccolithophore culture studies is mostly calculated from growth rate (obtained from daily cell densities plotted against time) and carbon quota (quantifiable through chemical analyses or from coccosphere/protoplast geometry); the latter usually measured only at the end of the experiment (Hoppe et al., 2011; Klintzsch et al., 2020; Langer et al., 2006; Langer, Nehrke, Thoms, & Stoll, 2009; Milner et al., 2016; Rosas‐Navarro et al., 2016; Zondervan et al., 2002). The underlying assumption of this method is that carbon quotas are constant over the course of the experiment, at least in the middle of the light phase (for a detailed discussion see Kottmeier et al., 2020). Although central to many studies, this assumption has rarely been explicitly tested, and few relevant datasets exist (Gerecht et al., 2015; Klintzsch et al., 2019; Kottmeier et al., 2020; Lenhart et al., 2016; Zondervan et al., 2002). Calculations based on non‐constant carbon quotas and/or non‐exponential growth lead to considerable errors (Gerecht et al., 2015; Kottmeier et al., 2020; Langer et al., 2012, 2013). Recently, two publications looked at a species never analyzed under increasing CO_2_ (and the resultant decreasing pH) before, despite its important role in CaCO_3_ production and export within the coccolithophore assemblage: *Helicosphaera carteri* (Bianco et al., 2025; Le Guevel et al., 2025). These studies demonstrated the ability of *H. carteri* (strain RCC1323 from the Roscoff Culture Collection) to maintain a stable ratio between particulate inorganic carbon (PIC) and particulate organic carbon (POC) under increasing CO_2_, suggesting that this species could make a stable contribution to the rain ratio under future climate changes (Bianco et al., 2025). Coccolith morphology is an important parameter in eco‐physiological terms (Langer et al., 2011, 2021). Bianco et al. (2025) highlighted a low morphological sensitivity of *H. carteri* to changes in carbonate chemistry compared to other heavily calcified species belonging to the *Calcidiscus* genus. To understand the eco‐physiological response of *H. carteri* to seawater acidification more fully, other important eco‐physiological parameters need to be taken into account, first and foremost growth rate and carbon production (Gafar et al., 2019a, 2019b; Langer, Nehrke, Probert, et al., 2009; Milner et al., 2016; Zondervan et al., 2002). In this study, we assessed the growth rate and PIC and POC production for a more comprehensive understanding of the response of *H. carteri* to increasing CO_2_. Since coccolithophore response patterns in typical OA experimental studies can display bell curves, it is necessary to use more than two CO_2_ levels if the aim is to detect the peak of the putative bell curve (Langer et al., 2006). Therefore, we added an intermediate CO_2_ level of 444 μatm CO_2_, falling between the previously used levels of 295 and 600 μatm (Bianco et al., 2025), to be able to describe in more detail the response pattern of *H. carteri* RCC1323. Moreover, we determined the daily carbon quota over the entire course of the experiments, aiming to verify whether carbon quotas remain constant during the exponential growth phase and to confirm the validity of the widely used assumption.

## MATERIALS AND METHODS

### Experimental setting and culture sampling

**FIGURE 1:**
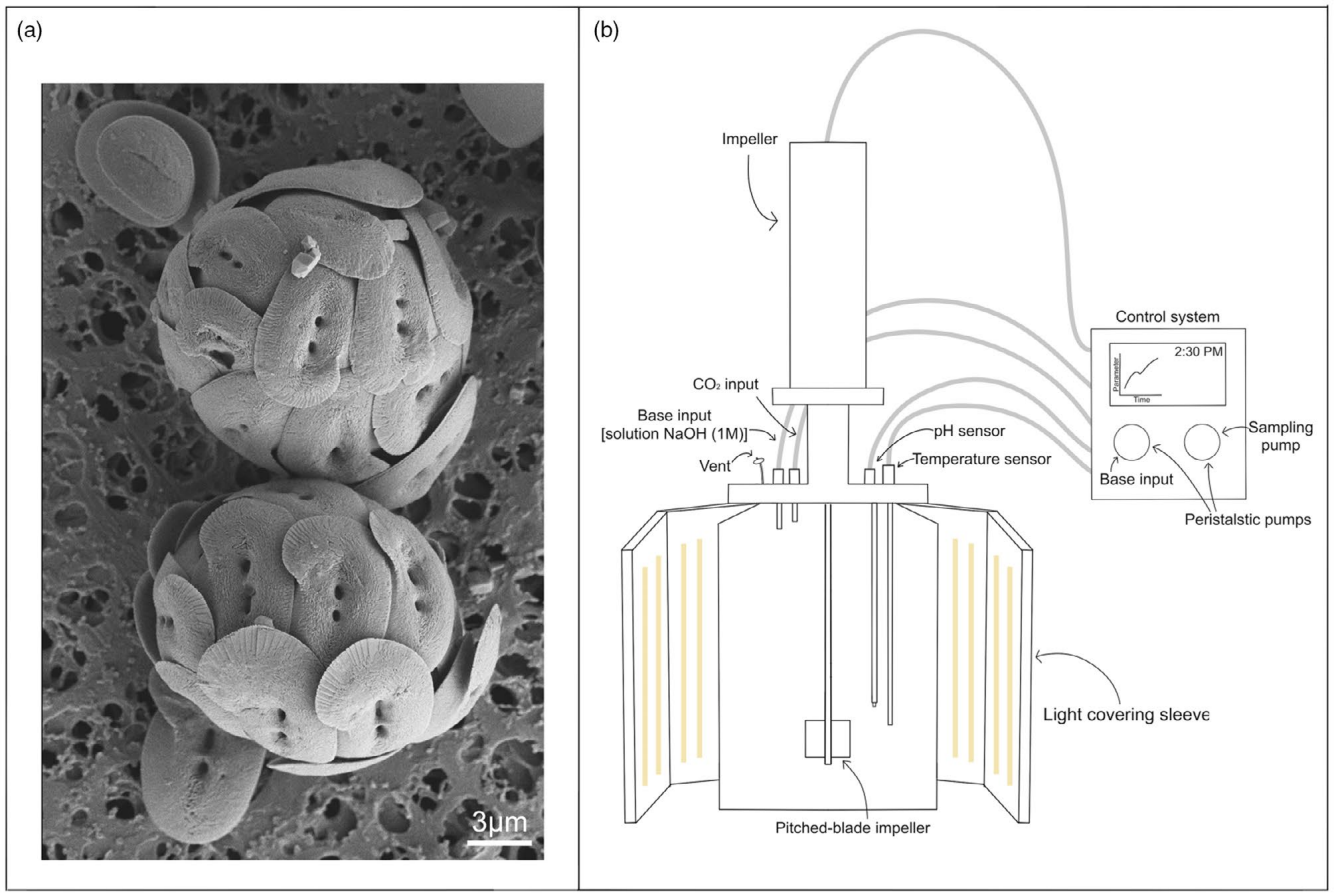
(a) Electron‐micrograph of *Helicosphaera carteri* coccospheres and coccoliths (magnification 7560×) taken at the final day of the experiment conducted at 295 μatm of CO_2_. The culture sample, 1 mL deposited on a cellulose acetate filter (pore size ∅ = 0.45 μm), was sputter‐coated with platinum before analysis with a scanning electron microscope (Tescan Mira3XMU) at the Department of Earth and Environmental Sciences at the University of Pavia (CISRiC‐Arvedi Laboratory). (b) Schematic representation of the Photobioreactor (PBR) and the experimental settings used to conduct the experiments.

A monospecific culture of *Helicosphaera carteri* (strain RCC1323; Figure 1a) from the Roscoff Culture Collection was grown simultaneously (see Table S1 for details) under three levels of CO_2_ at the National Institute of Oceanography and Applied Geophysics (OGS; Trieste, Italy). The CO_2_ concentrations used were ~295 μatm, mimicking the pre‐industrial levels (experiment 1); ~444 μatm, close to the Shared Socioeconomic Pathways (SSP) 1–2.6 scenario predicted for 2100 (experiment 2); and ~600 μatm, reproducing the SPP2‐4.5 scenario (Intergovernmental Panel on Climate Change [IPCC], 2023; experiment 3). In Ocean Acidification research the term “sensitive” refers to a change in, for example, growth rate or calcification rate in response to CO_2_ changes between ca. 200 and 1000 μatm (at approximately surface seawater dissolved inorganic carbon, DIC, and total alkalinity, TA; Hoppe et al., 2011). The term “sensitive” is not clearly defined, and higher CO_2_ levels have been used showing that, sometimes, effects only occur at “atypical” CO_2_ levels above 1000 μatm (Krug et al., 2011). Studies such as that performed at the atypic CO_2_ level by Krug et al. (2011) are worthwhile because they inform a wider set of physiological questions than the typical OA studies. In the context of OA and climate change, however, the typically chosen range of CO_2_ levels is most appropriate. Therefore, we chose typical values in the present study (from pre‐industrial levels to SSP 2–4.5 scenario‐predicted levels for 2100 by IPCC, 2023).

Experiments were run in triplicate, in photobioreactors (PBR) managed with Bioflex software using the dilute batch culture method (cf. Hoffmann et al., 2015). The experimental settings for the three CO_2_ levels were described in Bianco et al. (2025) and reported in Figure 1b, but the data about 444 μatm are newly presented here (Table S1). The inoculum, performed after acclimating the strain for ca. 11 generations, had an initial concentration of 1000 cells · mL^−1^. Cells were grown in dilute batch cultures to ensure a quasi‐constant carbonate chemistry over the course of the experiment (cf. Langer et al., 2006; LaRoche et al., 2010). Final cell densities of ca. 10,000 cells · mL^−1^ and a pH shift of, maximally, 0.03 indicate minor changes in carbonate chemistry over the course of the experiment (Langer et al., 2012, 2013). The pH levels were measured in continuum by the sensor inserted in the PBR (Hamilton PHI: 225; sensitivity: 57–59 mV · pH^−1^; frequency of measurements: 10 s). The collected data showed that the difference in pH from *t*_0_ to *t*_final_ was small, even for a typical dilute batch culture. We did not determine TA at the beginning of the experiment and consequently, there was some uncertainty about the exact carbonate chemistry change due to cell growth. Given our selection of final cell densities and the measured pH shift, however, this uncertainty is of minor importance. It is common practice not to report the full carbonate system at the start of the experiment (Hoffmann et al., 2015; Hoppe et al., 2011; Johnson et al., 2022; Langer et al., 2006, 2007; Langer & Benner, 2009; Langer & Bode, 2011; Langer, Nehrke, Probert, et al., 2009; Langer, Nehrke, Thoms, & Stoll, 2009). Changes in the carbonate chemistry due to cell growth, however, inevitably do occur, but even in experiments where considerable changes have been reported, the relative difference between treatments was still preserved; therefore, the experimental setup was suitable (Milner et al., 2016). An example of a more pronounced change in carbonate chemistry resulting from growing cells in batch mode (species‐specific maximum cell density at harvest day) was described in Langer et al. (2013). To effect such a change in a *Helicosphaera carteri* culture would require final cell densities an order of magnitude higher than the ones chosen here. Our experimental setup can, therefore, safely be considered suitable.

The carbonate system was calculated using the CO2SYS program (Lewis & Wallace, 1998) from temperature, salinity, TA, pH, phosphate, and silicate data. The data are reported in Table S2.

Every day, 4 mL of culture was sampled in the middle of the light phase, fixed with 40 μL of Lugol and used for cell counting. Cell counts were performed using an inverted optical microscope at 200× magnification. At least 200 cells were counted in each sample in three replicates using a Sedgwick‐Rafter counting chamber.

Growth curves were obtained by plotting cell density against time (days). The growth rate (μ) was determined from exponential regression (following Langer et al., 2006).

### Morphometric analyses on coccosphere and protoplast

Each day, 2 mL of culture were collected from each experiment for morphometric analyses. One milliliter of the sampled culture was fixed with prefiltered and neutralized formaldehyde (1.6% final concentration) and used for coccosphere morphometric analysis. The remaining volume (1 mL) was mixed with acidic Lugol solution (10 μL) to dissolve the coccoliths while preserving the protoplast, for analyzing the protoplast geometry.

Coccosphere size (∅) and protoplast size (Θ), along with aspect ratio (AR) and roundness (RD), were measured by capturing images of over 50 coccospheres and 50 protoplasts per replicate, for a total of ~7200 images. All the images were collected at 400× magnification with an inverted Leica microscope (CMS‐D35578) and a Leica camera (CH‐9435) and analyzed with ImageJ software (Rueden et al., 2017) through a customized macro (https://github.com/mbordiga/Coccoliths). For morphometric measurements, only cells positioned on their side were selected, ensuring that their elliptical shape was visible.

*Helicosphaera carteri* cellular particulate organic carbon (POC_geometry_) was calculated daily (i.e., from *t*_0_ to *t*_final_) from protoplast size, using the method of Menden‐Deuer and Lessard (2000):

(1) $$\frac{\mathrm{POC}\;\left[\mathrm{pg}\right]}{\text{cell}}\equiv a\times V_{\text{cell}}^{b}$$

The protoplast volume is denoted by *V*_cell_, with *a* and *b* representing constants that vary based on the species examined (for *Helicosphaera carteri a* = 0.216 and *b* = 0.939; Menden‐Deuer & Lessard, 2000). Protoplast volume, expressed in μm^3^, was determined using the approach outlined by Sun and Liu (2003):

(2) $$V_{c\mathrm{ell}}^{b}=\left(\pi /6\right)d^{2}\times h$$

where *d* and *h* represent the short and the long‐axes cell diameters in μm, respectively.

A comprehensive list of the new morphometric data discussed in this work and those already published in Bianco et al. (2025) is presented in Table S1.

To estimate the organic and inorganic carbon production rates of *Helicosphaera carteri* at *t*_final_ (*t*_*f*_), data on the cellular POC calculated in this study and PIC presented in Bianco et al. (2025), were multiplied by the growth rate of the species (μ):

(3) $$\mathrm{pg}\;C\cdot {\text{cell}}^{-1}\cdot {\mathrm{day}}^{-1}=\mu \times \left(\text{cellular carbon content}\right)$$

For the samples from experiment 2, it was not possible to estimate the cellular inorganic carbon quota and, consequently, the PIC production because the samples for assessing the number of coccoliths per coccosphere under scanning electron microscopy (SEM) were lost.

Since AR and RD of both protoplasts and coccospheres showed a strong negative correlation (−0.99, *p*‐value < 0.0001), only the RD data have been discussed in this work. Changes in ∅, Θ, RD, and PIC and POC quotas and production rates within and among the experiments were analyzed using paired and unpaired *t*‐tests at a 0.05 confidence level on GraphPad Prism (version 9.05 for MacOS; GraphPad Software, Inc., United States).

### Chemical analyses

#### Dissolved inorganic carbon (DIC) analysis

For DIC analysis, samples were collected in duplicate (40 mL each) on the final day of each experiment by filtering the culture over pre‐combusted (450°C for 4 h) Whatman GF/F glass‐fiber filters (0.7‐μm nominal pore size). During the sample collection, the gas exchange with the atmosphere was reduced, and to inhibit biological activity, samples were poisoned with a 50% mercuric chloride solution (HgCl_2_, Sigma Aldrich), prepared by diluting 1:1 (w:w) a saturated HgCl_2_ solution with milliQ water. The samples were then stored at 4°C until analysis. Dissolved inorganic carbon was measured using a Shimadzu TOC‐V CSH analyzer as described in De Vittor et al. (2016). Samples were automatically injected into the instrument port and acidified with H_3_PO_4_ (25%), and the CO_2_ generated was carried to a nondispersive infrared detector for the DIC measurements. The accuracy of the results (± 2.0 μmol · kg^−1^) was guaranteed by the analysis of certified reference material (CRM, Batch #107) and by biannual participation in the international intercalibration exercises (Wepal‐Quasimeme Laboratory Performance Study, Wageningen Research, Netherlands).

#### Total alkalinity (TA) analysis

At the final day of each experiment, 100 mL samples of culture were filtered through pre‐combusted glass‐fiber filters (Whatman GF/F; nominal pore size of 0.7 μm) and collected in 250‐mL acid‐washed borosilicate flasks (Pyrex). To stop biological activity, 100 μL of saturated HgCl_2_ (0.75 mol · L^−1^ in milliQ) was added before sealing and storing the samples at 4°C in the dark. Total alkalinity was measured through a two‐stage, potentiometric, open cell titration according to standard operating procedure (SOP) 3b (Dickson et al., 2007) with a G20 titration unit equipped with a glass pH probe DGİ115‐SG and controlled by LabX data‐acquisition software (all Mettler‐Toledo). The HCl titrant solution (0.1 mol · kg^−1^) was prepared in a NaCl background to match the ionic strength of the media and was calibrated using the CRM (Andrew Dickson, Scripps, California, United States, batch 107). Density of the titrant solution was evaluated hydrostatically with a Mohr balance (Orma Eurotek, Italy). Total alkalinity was calculated from titration data using a non‐linear least squares method via an in‐house software program, developed at OGS as described in SOP 3 (Dickson & Goyet, 1994) and adapted to function with the LabX software. The accuracy and precision of the TA measurements on CRM were determined to be within ±2.0 μmol · kg^−1^.

## RESULTS

### *Helicosphaera carteri* protoplast and coccosphere geometry

#### Protoplast geometry

**FIGURE 2:**
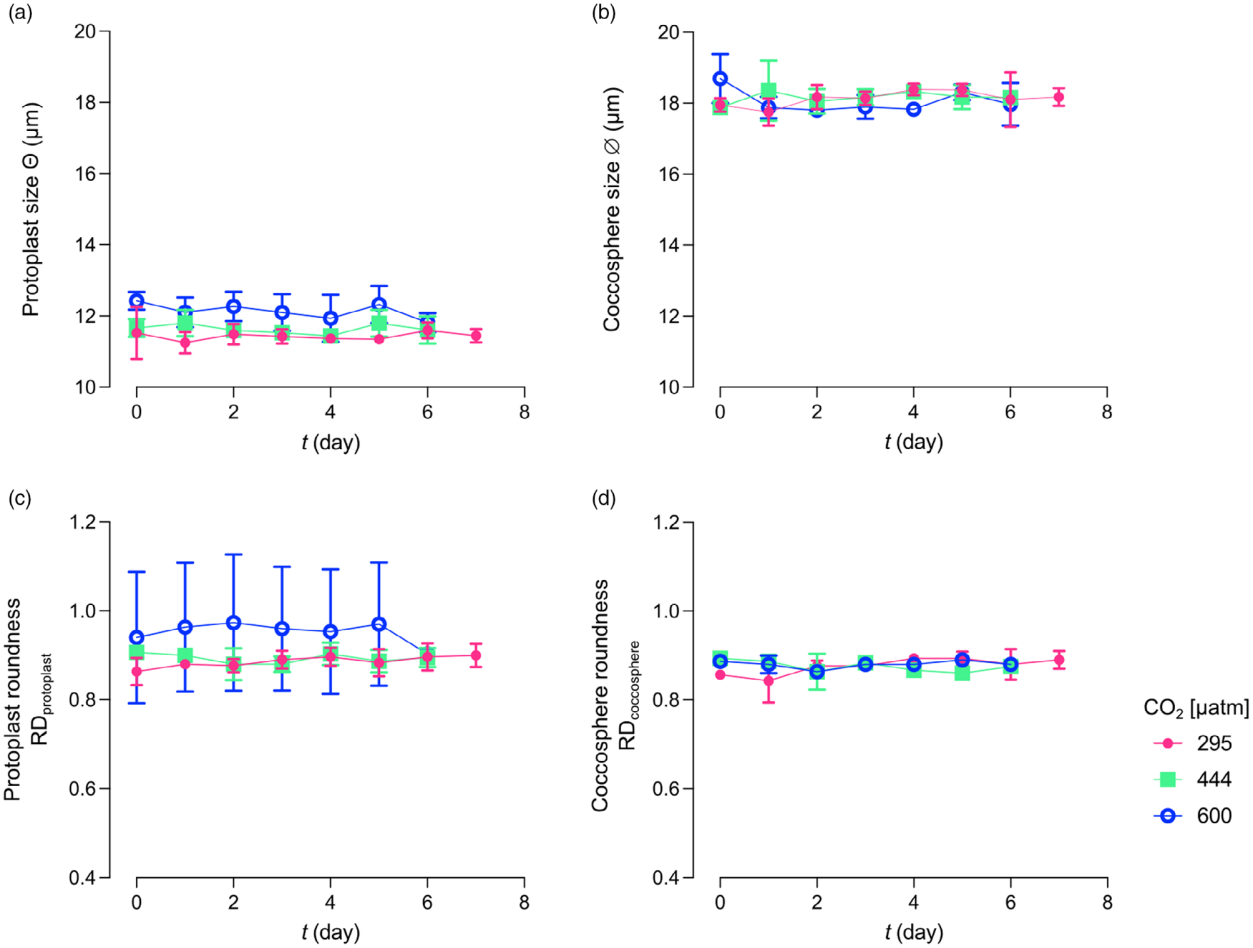
Morphometrical data from the experiments on *Helicosphaera carteri*. Protoplast size (a) and roundness (RD; (b)); coccosphere size (c) and roundness (d) from *t*_0_ to *t*_f_, at the three different CO_2_ levels. The reported values represent the averages of the three replicates. Error bars show the standard deviation of the three replicates per each experiment.

The variation in protoplast size (Θ) did not show a consistent trend across the three different experiments, and it did not change significantly from the beginning to the end of each experimental condition (Figure 2a; *t*‐test, *p* > 0.05). Protoplast roundness (RD_protoplast_) did not show any significant variation from the beginning to the end of each experiment (*t*‐test, *p* > 0.05; Figure 2b; Table S3).

#### Coccosphere geometry

*Helicosphaera carteri* coccosphere size (∅) did not show significant variation from the beginning to the end of all three experiments, with a fluctuation of the coccosphere size ranging from a minimum of 17.75 ± 0.38 μm recorded at *t*_1_ of experiment 1 to a maximum of 18.70 ± 0.69 μm at *t*_0_ of experiment 3 (Figure 2c; Table S3). The minimum and the maximum ∅ values occurred at *t*_1_ and *t*_4_, respectively, for the experiment at 295 μatm, during *t*_0_ and *t*_1_ at 444 μatm and during *t*_2_ and *t*_0_ at 600 μatm (Figure 2c; Table S3). A non‐consistent variation in coccosphere roundness (RD_coccosphere_) was also observed among the three experiments (*t*‐test, *p* > 0.05; Figure 2d; Table S3).

### POC_geometry_ quota

**FIGURE 3:**
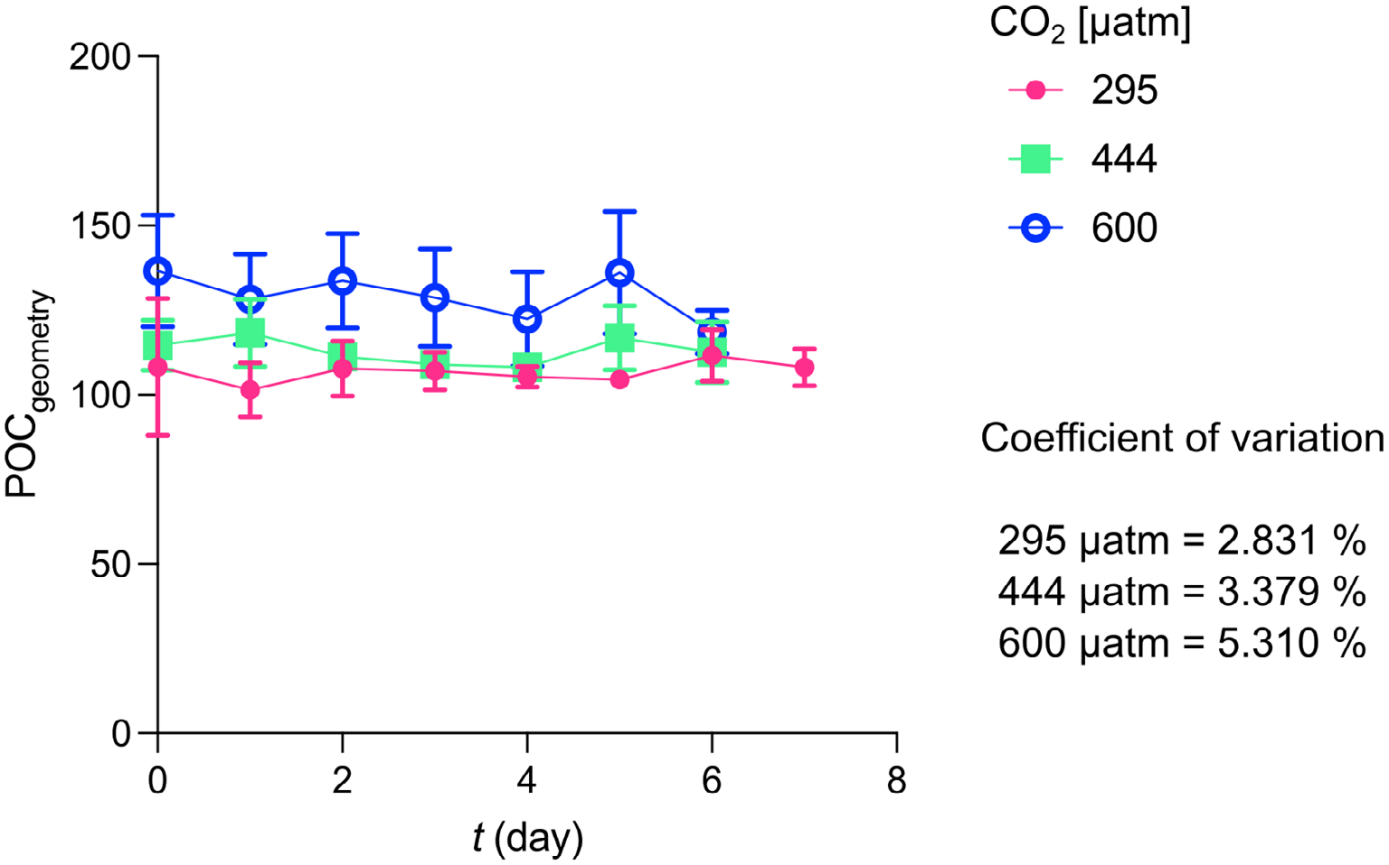
Particulate organic carbon quotas from *t*_0_ to *t*_*f*_, at the three different CO_2_ levels. The reported values represent the averages of the three replicates. Error bars show the standard deviation of the three replicates per each experiment.

The POC quota calculated from protoplast size did not show a consistent trend across the three experiments from *t*_0_ to *t*_f_ (Figure 3; Table S3). A non‐significant variation in POC_geometry_ quota was observed at all the CO_2_ levels tested (*t*‐test, *p* > 0.05).

### Growth rate and particulate organic carbon (POC) and particulate inorganic carbon (PIC) quotas, production, and rates

**FIGURE 4:**
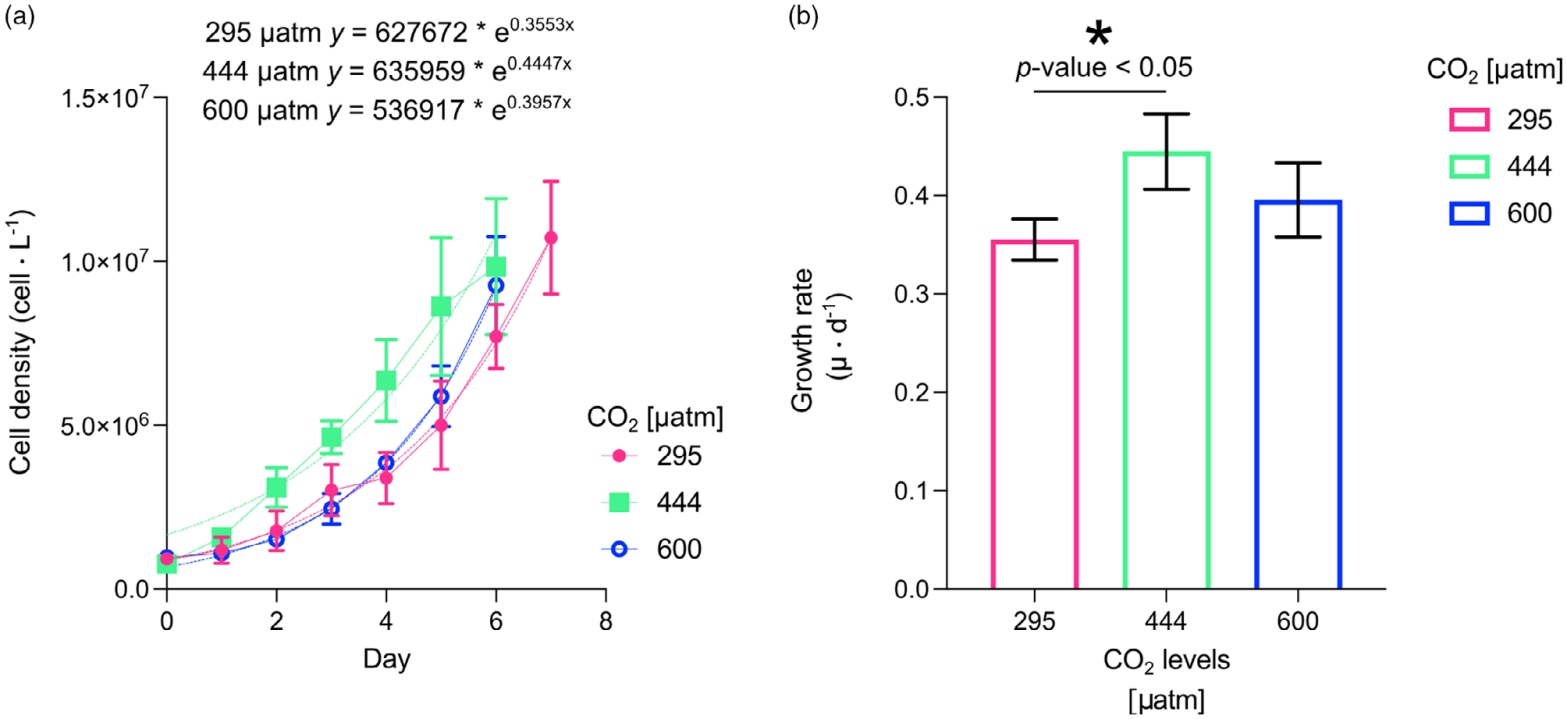
Growth curves (a) and growth rates (b) of *Helicosphaera carteri* under the three different CO_2_ levels. The error bars represent the standard deviation of three replicates per each experiment. The asterisk indicates when the change is statistically significant (*p* < 0.05).

In our experiments with *Helicosphaera carteri*, growth rates (μ · day^−1^) changed with increasing CO_2_ / decreasing pH in a non‐linear way. The maximum growth rate of 0.44 ± 0.04 μ · day^−1^ was obtained at 444 μatm. At 295 and 600 μatm, growth rates were 0.36 ± 0.02 μ · day^−1^ and 0.40 ± 0.04 μ · day^−1^ respectively (Table 1; Figure 4). Although the increased growth rate from 295 to 444 μatm showed statistical significance (*t*‐test *p* = 0.0238; Table 1), there was no significant variation between the intermediate and the highest levels of CO_2_ (*t*‐test, *p* > 0.05; Table 1).

**TABLE 1:** Eco‐physiological responses of *Helicosphaera carteri* to changes in pCO_2_.

Experiment	1	2	3
CO_2_ (μatm)	294.56	**444.48**	601.51
SD	17.84	**84.45**	59.74
**Growth rate (μ ⋅ day** ^**−1**^)	**0.36**	**0.44**	**0.40**
**SD**	**0.03**	**0.04**	**0.04**
Cellular POC content (pg POC ⋅ cell^−1^)	108.14	**110.40**	118.51
*SD*	5.42	**7.39**	6.41
**Cellular POC production (pg POC ⋅ cell** ^**−1**^ **⋅ day** ^**−1**^)	**38.45**	**49.10**	**46.95**
***SD***	**3.52**	**5.29**	**5.95**
Cellular PIC content (pg PIC ⋅ cell^−1^)	151.86	/	149.47
*SD*	4.23	/	9.49
**Cellular PIC production (pg PIC ⋅ cell** ^**−1 ⋅**^ **day** ^**−1**^)	**53.95**	**/**	**59.14**
***SD***	**2.99**	**/**	**8.60**
PIC:POC	1.37	/	1.27
*SD*	0.07	/	0.13

*Note*: Data reported are the averages obtained from the replicates of each experiment from Bianco et al. (2025) and from this work (in bold).

Abbreviation: *SD*, standard deviation.

The organic carbon content in *Helicosphaera carteri* did not vary significantly among the three experiments (*t*‐test, *p* > 0.05) with a minimum value of 108.14 ± 5.42 pg. · cell^−1^ at 295 μatm and a maximum value of 118.51 ± 6.41 pg. · cell^−1^ at 600 μatm (Table 1; Figure 4a).

Particulate organic carbon production significantly changed (*t*‐test, *p* < 0.05) between 295 μatm (38.45 ± 3.52 pg. · cell^−1^ · day^−1^) and 444 μatm (49.10 ± 5.29 pg. · cell^−1^ · day^−1^), but it did not show any significant variation between the intermediate and the highest CO_2_ concentrations (*t*‐test, *p* > 0.05; Table 1; Figure 4c). Inorganic carbon production did not change significantly (*p* > 0.05; Table 1; Figure 4d) from the lowest CO_2_ level (53.95 ± 2.99 pg. · cell^−1^ · day^−1^) to the highest (59.14 ± 8.60 pg. · cell^−1^ · day^−1^). As already reported in Bianco et al. (2025), PIC:POC did not show any significant variations under increasing CO_2_ (Table 1; Figure 4e).

## DISCUSSION

### Protoplast and coccosphere geometry variations along culture experiments

To estimate the production of organic and inorganic carbon in coccolithophores under experimental conditions, data on cellular PIC and POC from *t*_final_ and growth rate are usually used (Klintzsch et al., 2019; Langer, Nehrke, Probert, et al., 2009; Šupraha et al., 2015). The calculation is based on the assumption that PIC and POC quotas do not change over the course of the experiments. Here, to test whether the organic carbon cellular content in *Helicosphaera carteri* did vary along the experiment, we reconstructed the cellular POC quota of the species from protoplast size on a daily basis. The data showed that the POC quota did not vary significantly during the experiments, even if when *H. carteri* was grown under different CO_2_ concentrations/pH values, confirming the validity of the method used to calculate production. This method presupposes both constant quotas and exponential growth. Our dilute batch approach met both requirements (Figures 1 and 2). When transitioning from exponential to stationary growth phase, coccolithophore cultures display non‐constant, usually increasing, quotas, which render the determination of production difficult, if not impossible (Langer et al., 2012, 2013; Lenhart et al., 2016; Oviedo et al., 2014). Exponential growth avoids these problems, but sampling for quota determination needs to be conducted at a fixed point in the light:dark cycle, ideally in the middle of the light phase (this study; Kottmeier et al., 2020; Langer et al., 2013; Zondervan et al., 2002).

We can, therefore, only speculate whether PIC quota would show the same constancy as POC quota. Given the remarkable constancy of the PIC:POC ratio in *Helicosphaera carteri* we hypothesize that the PIC quota is also constant over the course of the experiment (Figure 4).

Apart from methodologically relevant carbon quotas, roundness, indicative of the general physiological status of the cells, was also constant over the course of the experiment (Figure 2). Whereas protoplast roundness might be of limited value when assessing the general physiological status, coccosphere roundness has proved telling (Bianco et al., 2025). The constancy of coccosphere roundness, both over the course of the experiment and between treatments, pointed to a physiological state that was unaffected by both dilute batch cell densities and carbonate chemistry. Although this inference is supported by morphological data and PIC:POC ratios, it is qualified by rate data as detailed in the next section.

### *Helicosphaera carteri* under varying CO_2_ levels

**FIGURE 5:**
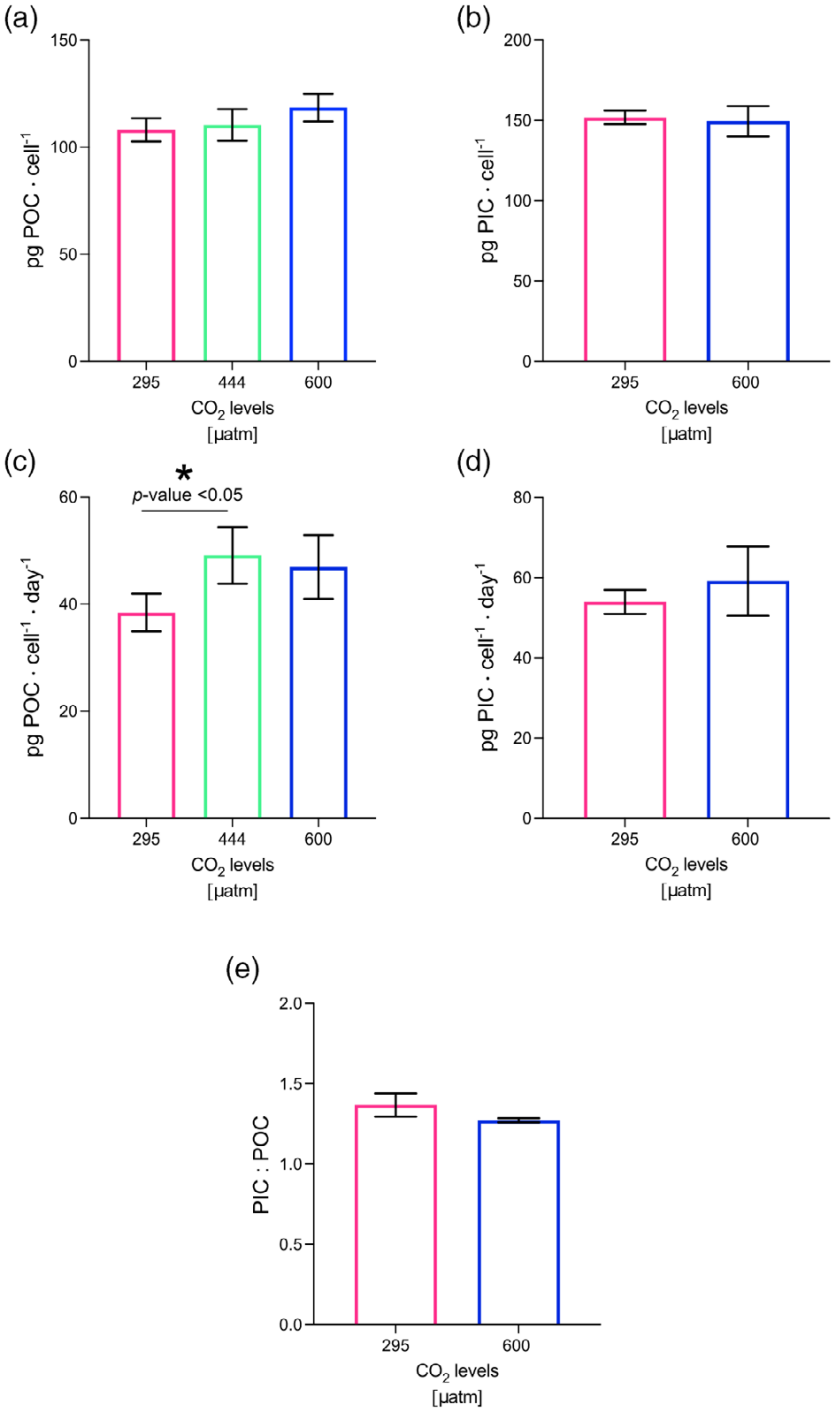
Particulate organic (a) and inorganic carbon quotas (b) and production (c, d), and PIC:POC ratio (e) under the three different experimental conditions. Error bars show the standard deviation of the three replicates per each experiment. The asterisk indicates when the change is statistically significant (*p* < 0.05).

**FIGURE 6:**
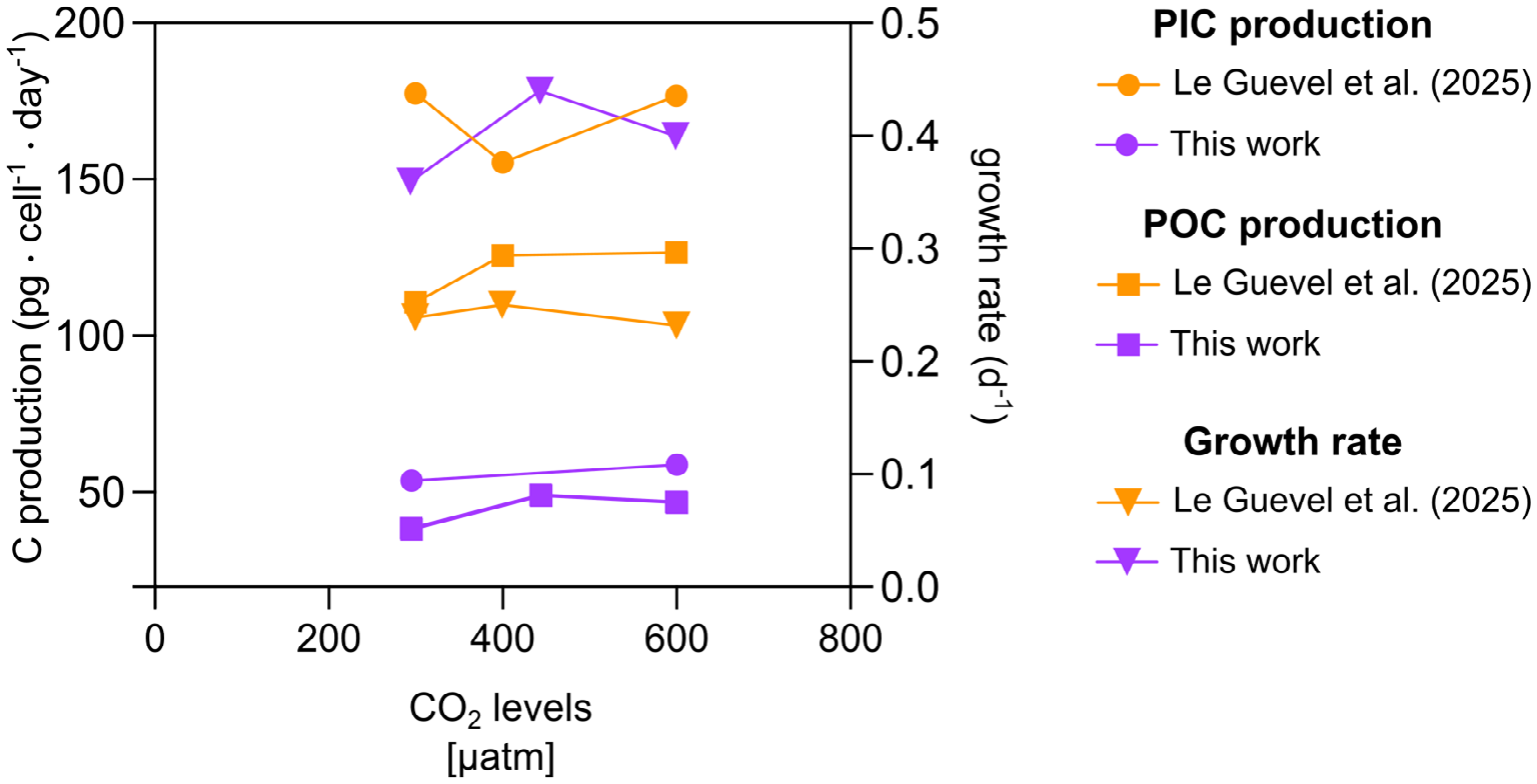
Comparison of *Helicosphaera carteri* growth and particulate inorganic carbon (PIC) and particulate organic carbon (POC) production rates from this work and Le Guevel et al. (2025), obtained using the free license software XyScan (Version 4.6.6).

So far, only a few studies have been conducted on *Helicosphaera carteri* under experimental conditions, and only two have looked at the response to increasing CO_2_ (Bianco et al., 2025; Gussone et al., 2007; Le Guevel et al., 2025; Sheward et al., 2017; Stoll et al., 2002; Šupraha et al., 2015; Šupraha & Henderiks, 2020). Among the previous studies, Šupraha and Henderiks (2020) tried to reconstruct the response of *Helicosphaera* under decreasing CO_2_ levels by analyzing its fossil record of the last 15 million years. The authors observed a consistent decrease in *Helicosphaera* coccolith and cell size over time (from the Miocene to the Pleistocene) across various levels—morpho‐specific, community, and evolutionary—as had been previously observed for other species belonging to the Noelaerhabdaceae group (Suchéras‐Marx & Henderiks, 2014). The authors showed that unlike lightly calcified species, *Helicosphaera* maintained a relatively stable PIC:POC ratio over time, despite the size reduction. They associated this lack of variance in biogeochemical output (combined with conservative morphology and low phenotypic plasticity) with the obligate calcifier nature of *Helicosphaera*, a trait also shared by the genera *Calcidiscus* and *Coccolithus* (Walker et al., 2018; Ian Probert, pers. comm., 2024; own observation, 2024). The constant PIC:POC ratio in the fossil record has also been mirrored by a constant PIC:POC in carbonate chemistry manipulation experiments (Figures 5 and 6; Bianco et al., 2025; Le Guevel et al., 2025). This correspondence seems to support the idea that obligate calcifiers need to carefully balance their DIC allocation despite changing physiological rates. Although we can only speculate that over geological timescales growth rates and organic carbon production may have varied, we know that these rates changed in our experiment with changing carbonate chemistry (Figures 3 and 4). These rate changes did not affect inorganic carbon allocation, however, as evidenced by the stable PIC:POC ratio.

Is the obligate calcifier nature of *Helicosphaera carteri* a sufficient explanation for the stable PIC:POC despite carbonate chemistry induced rate changes? We propose that a stable PIC:POC ratio in obligate calcifiers is an indicator of insensitivity to carbonate chemistry changes. *Helicosphaera carteri* (RCC1323), in this sense, behaves like an insensitive *Coccolithus braarudii* strain RCC1200 (constant PIC:POC and morphology), whereas a more sensitive *Coccolithus braarudii* strain PLY182g showed variable PIC:POC alongside altered coccolith morphology (Kottmeier et al., 2022; Langer et al., 2006). By contrast, the obligate calcifier *Calcidiscus leptoporus* is sensitive to carbonate chemistry, and it showed altered PIC:POC as well as morphology in response to carbonate chemistry changes (Diner et al., 2015; Langer et al., 2006; Langer & Bode, 2011). Considering that all of these strains feature similar and relatively high PIC:POC (>1, typically ca. 1.3–2), sensitivity to carbonate chemistry cannot be inferred from the PIC:POC ratio alone. When comparing different species over a large range in PIC:POC, POC is nevertheless a good predictor of sensitivity (Gafar et al., 2019b). It is also important to keep in mind that sensitivity can be differently assessed, by considering, for example, morphology, PIC:POC, growth rate, and so on. *Helicosphaera carteri* is sensitive with respect to growth rate and organic carbon production but not with respect to PIC production, PIC:POC, and morphology (Figures 4 and 5; Bianco et al., 2025). In eco‐physiological terms, growth rate is certainly a relevant parameter, and morphology might be similarly relevant (Langer et al., 2011, 2021). Therefore *H. carteri* is not as insensitive as suggested solely based on morphology, PIC:POC, and roundness (Bianco et al., 2025). Although our coupled C‐system did not allow us to determine the parameters of the C‐system‐causing effects, it would be interesting to look in more detail at the effects of carbonate chemistry on growth rate and carbon production.

We have shown that *Helicosphaera carteri* increases its POC production and growth rates from low to intermediate CO_2_ concentrations and keeps a stable PIC production as the CO_2_ level changes (Figure 6). Growth rate and POC production were not affected by a CO_2_ increase from the intermediate CO_2_ level to 600 μatm. This response pattern is compatible with the substrate‐limitation–proton‐inhibition concept (Bach et al., 2011, 2015). At 295 μatm of CO_2_, the lower growth rate and POC production determined here seemed to suggest CO_2_ limitation. At 444 μatm, CO_2_ rates seemed to be saturated because there was no further increase in rates at 600 μatm CO_2_. There also seemed to be no proton inhibition because there was no significant rate decrease at 600 μatm CO_2_. Alternatively, it cannot be excluded that there would be a further CO_2_‐fueled rate increase from 444 to 600 μatm CO_2_ if there was no simultaneous proton inhibition canceling the effect.

However, it is important to note that this rate response under a similar gradient of increasing CO_2_ was not observed in Le Guevel et al. (2025), in which the growth rate of *Helicosphaera carteri* was lower compared to that in our experiments (average μ = 0.24 · day^−1^ in Le Guevel et al., 2025; average μ = 0.40 · day^−1^ in this work), probably due to differences in the experimental settings. Among these, the temperature difference could play a key role in the development of dissimilar responses in the same strain. Indeed, although in our work *H. carteri* was grown under 19°C, in Le Guevel et al. (2025) the species was grown at 15°C. The higher temperatures characterizing our experiments may have led to faster growth of *H. carteri*, despite the similar CO_2_ concentrations. Temperature modulation of carbonate chemistry effects has been described in coccolithophores, supporting this interpretation (Johnson et al., 2022; Milner et al., 2016; Sett et al., 2014).

The observation of limiting conditions in *Helicosphaera carteri* at CO_2_ levels (9.78 μmol · kg^−1^; Bianco et al., 2025) higher than those identified as limiting for *Gephyrocapsa huxleyi* (~7.5 μmol · kg^−1^; Bach et al., 2013) could be related to species‐specific differences, in turn, related to size. The bigger *H. carteri* presumably has a bigger diffusive boundary layer than *Gephyrocapsa huxleyi* and, therefore, experiences lower CO_2_ concentrations at the cell surface (see also discussion in Langer & Bode, 2011).

Another important aspect to consider is the range of CO_2_ at which *Helicosphaera carteri* was grown in this work. Usually, to test coccolithophores' responses to OA in culture experiments, a wider CO_2_ range is used (in general from ~180 to at least 800 ppm of CO_2_). We can only infer what could have happened to the species at lower and higher CO_2_ concentrations compared with this work, based on the results presented here and by Le Guevel et al. (2025), who despite using a different experimental setting, grew the species from 200 to 1400 ppmv of CO_2_.

According to the results obtained in this work, we can assume that if *Helicosphaera carteri* had been grown under even lower CO_2_ concentrations, its growth and organic carbon production rates might have continued to decrease. Moreover, based on the findings from Bach et al. (2011, 2013, 2015) and Sett et al. (2014) on other coccolithophore species, a decrease in its calcification rate could be expected too.

With regard to the species' responses under CO_2_ concentrations higher than 600 μatm, Le Guevel et al. (2025) documented for *Helicosphaera carteri* the maintenance of a stable growth rate and PIC:POC under the entire CO_2_ range tested, parallel to a decrease in coccosphere size. However, as already discussed above, in Le Guevel et al. (2025) the strain RCC1323 behaved in a different way compared to our experiments, due to the difference in experimental settings. The higher growth rate of the species observed in our experiment could be critical for determining a different response of the species under similar CO_2_ concentrations and could be related to a negative response under decreasing pH/increasing CO_2_. As already observed for other species (e.g., *Gephyrocapsa huxleyi*, *Gehyra oceanica*, *Coccolithus braarudii*, *Calcidiscus leptoporus*), *H. carteri* would reach an optimum beyond which its capability to calcify, grow, and perform photosynthesis would decrease. However, to verify at which CO_2_ concentration this will happen, new culture experiments are required.

### Coccolithophores versus CO_2_ : A comparison among different species and strains

In the past few decades significant efforts have been made to try to define coccolithophore responses to increasing CO_2_, and different, sometimes contrasting, results have been reported. After identifying variations in responses among species belonging to the same group, or even among strains within the same morphotype, it has been suggested that the response/sensitivity to increasing CO_2_ of a particular species or strain has a genetic basis (Langer, Nehrke, Probert, et al., 2009). A link between the sensitivity to changes in carbonate chemistry and PIC:POC has also been hypothesized (Bach et al., 2015; Diner et al., 2015; Gafar et al., 2019b). In this context, a high sensitivity to increasing CO_2_ was suggested for *Helicosphaera carteri* (Gafar et al., 2019b). Here, to evaluate whether *H. carteri* is one of the more sensitive species, we compared the variations in its growth rate, organic and inorganic carbon quotas and production, and PIC:POC with data from works conducted on other species under increasing CO_2_ concentrations (Table 2). The results showed that *H. carteri* appears to be less sensitive to increasing CO_2_ / decreasing pH than anticipated. In terms of growth rate, a lower sensitivity was observed for *H. carteri* compared with five strains of *Gephyrocapsa huxleyi* (B92/11, RCC1256, RCC1212, RCC1216 and PLY837) and strains RCC1168 of *Calcidiscus quadriperforatus* and RCC1141 and RCC1130 of *Calcidiscus leptoporus* as these species have shown a decrease in their growth with increasing CO_2_ (see Table 2 for references). The strains RCC1238, RCC1266, and AC472 of *Gephyrocapsa huxleyi* and the species *Calcidiscus* *leptoporus* (AC365), *Cocoolithus braarudii* (RCC1198) *Gehyra oceanica* (RCC1314), and *Chrysotila carterae* (PLY406) have shown similar sensitivities compared to *H. carteri*, without showing any significant variations in growth rate under rising CO_2_ levels (Chauhan & Rickaby, 2024; Fiorini et al., 2011; Langer et al., 2006, Langer, Nehrke, Probert, et al., 2009; Langer, Nehrke, Thoms, & Stoll, 2009; Le Guevel et al., 2025; Zhang et al., 2023). The strains RCC1216 and NZEH of *Gephyrocapsa huxleyi* and RCC1200 of *Coccolithus braarudii* have been reported to show either a decrease or no changes in growth rates in different studies. Strain RCC1198 showed equal or greater sensitivity compared to *H. carteri* (Table 2).

**TABLE 2:** Responses of different species and strains of coccolithophores under increasing CO_2_ in culture experiments.

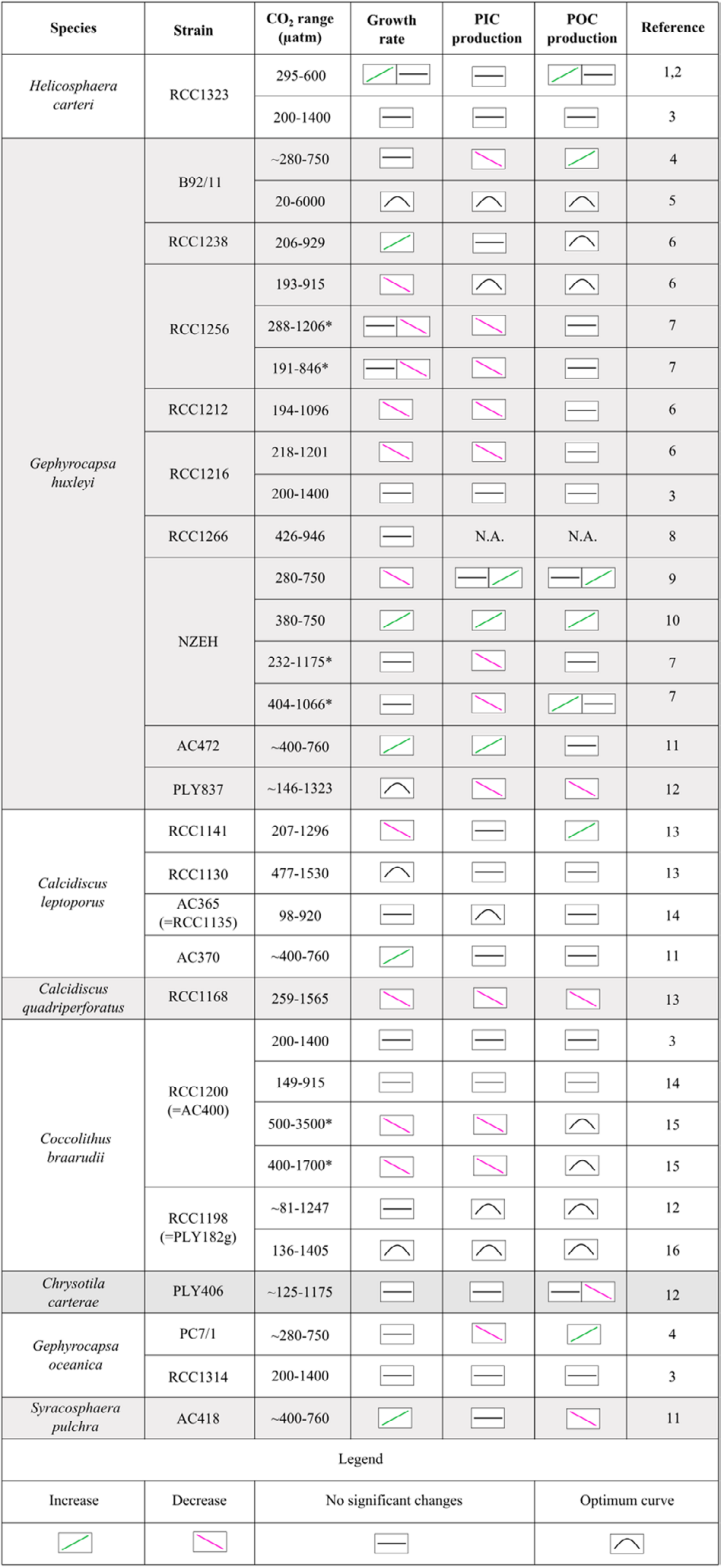

*Note*: Data shown include growth rate and particulate inorganic carbon (PIC) and particulate organic carbon (POC) production rates. Reference column indicates that data are from: (1) this work; (2) Bianco et al. (2025); (3) Le Guevel et al. (2025); (4) Riebesell et al. (2000); (5) Bach et al. (2011); (6) Langer, Nehrke, Probert, et al. (2009); (7) Hoppe et al. (2011); (8) Zhang et al. (2023); (9) Iglesias‐Rodriguez et al. (2008); (10) Shi et al. (2009); (11) Fiorini et al. (2011); (12) Chauhan and Rickaby (2024); (13) Diner et al. (2015); (14) Langer et al. (2006); (15) Krug et al. (2011); (16) Kottmeier et al. (2022). The asterisks indicate two types of carbonate chemistry manipulations in the same species by the same author. N.A.: data not available. Data reported from Chauhan and Rickaby (2024) refer to the initial conditions.

Regarding the influence of changes in carbonate chemistry on PIC quotas and/or production a higher sensitivity compared to *Helicosphaera carteri* has been observed for most *Gephyrocapsa huxleyi* strains, *Calcidiscus leptoporus* AC365, *Calcidiscus quadriperforatus* RCC1168, *Cocoolithus braarudii* RCC1198, *Gehyra oceanica* PC7/1, and *Syracosphaera pulchra* (see Tables 2 and 3). As for growth rate, *Coccolithus braarudii* RCC1200 and *Gephyrocapsa huxleyi* NZEH have shown variable and sometimes contrasting responses in different studies (Tables 2 and 3). Among these species, *Gephyrocapsa huxleyi* B92/11, RCC1212, and 1266; *Calcidiscus leptoporus* RCC1141 and AC365; and *Gehyra oceanica* PC7/1 have shown a higher sensitivity in terms of PIC:POC (Table 3). The strains RCC1256, 1216, and NZEH of *Gephyrocapsa huxleyi* and RCC1200 of *Coccolithus braarudii* showed higher or similar sensitivity compared to *H. carteri* (PIC:POC decreased or remained unchanged). A similar response in terms of PIC:POC (not varying with CO_2_ increase) compared to *H. carteri* was also observed for *Gephyrocapsa huxleyi* RCC1238 and PLY827, *Calcidiscus leptoporus* AC370, *Gehyra oceanica* RCC1314, *Chrysotila carterae, Syrac. pulchra*, and *Calcidiscus quadriperforatus* (Table 3).

**TABLE 3:** Responses of different species and strains of coccolithophores under increasing CO_2_ in culture experiments.

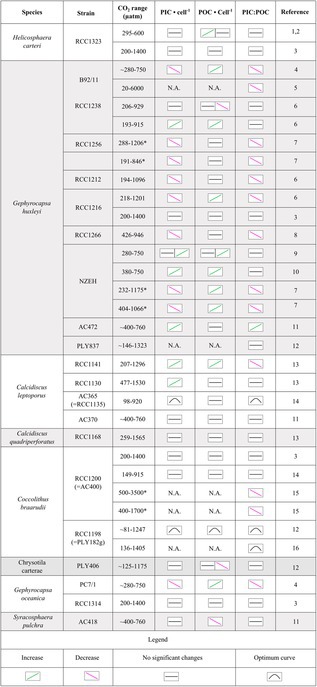

*Note*: Data shown include particulate inorganic carbon (PIC) and particulate organic carbon (POC) quotas and PIC:POC ratio. Numbers in the reference column indicate data are from: (1) this work; (2) Bianco et al. (2025); (3) Le Guevel et al. (2025); (4) Riebesell et al. (2000); (5) Bach et al. (2011); (6) Langer, Nehrke, Probert, et al. (2009); (7) Hoppe et al. (2011); (8) Zhang et al. (2023); (9) Iglesias‐Rodriguez et al. (2008); (10) Shi et al. (2009); (11) Fiorini et al. (2011); (12) Chauhan and Rickaby (2024); (13) Diner et al. (2015); (14) Langer et al. (2006); (15) Krug et al. (2011); (16) Kottmeier et al. (2022). The asterisks indicate two types of carbonate chemistry manipulations in the same species by the same author. N.A.: data not available. Data reported from Chauhan and Rickaby (2024) refer to the initial conditions.

In contrast to *Helicosphaera carteri*, *Gephyrocapsa huxleyi* strain RCC1238 and *Syracosphaera pulchra* AC418 showed decreases in POC quotas with increasing CO_2_, indicating higher sensitivity to OA. Note, however, that the POC quota has limited physiological meaning compared to rates. A constant quota can be the result of a change in both production and growth rate. Finally, higher sensitivity in POC production compared to *H. carteri* has been observed in *S. pulchra* AC418, *Coccolithus braarudii* RCC1198, *Gephyrocapsa huxleyi* RCC1238 and PLY837, and *Calcidiscus quadriperforatus* RCC1168, also, in some studies, for *Gephyrocapsa huxleyi* B92/11. Higher or similar sensitivity has been documented for *Gephyrocapsa huxleyi* RCC1256 and *Coccolithus braarudii* 1200 (see Table 2). However, most of these observations were based on a wider range of CO_2_ concentrations, specifically on even higher CO_2_ levels than 600 μatm. A direct comparison of *H. carteri* with the other species grown under similar CO_2_ ranges (from 280 to 600 μatm of CO_2_) confirmed the higher sensitivity of the strains B92/11 of *Gephyrocapsa huxleyi*, RCC1198 of *Coccolithus braarudii*, and AC365 of *Calcidiscus leptoporus* (*c.f*. Bach et al., 2011; Chauhan & Rickaby, 2024; Kottmeier et al., 2022; Langer et al., 2006; Tables 2 and 3). However, due to the high variability in response patterns observed between different species and strains (Hoppe et al., 2011; Langer et al., 2006; Langer, Nehrke, Probert, et al., 2009), new studies considering different *H. carteri* strains will be fundamental for better understanding responses to future changes in seawater chemistry.

## CONCLUSIONS

The evidence reported in this work on cell geometry collected on a daily basis has revealed that the POC quota of *Helicosphaera carteri* does not change over the course of a dilute batch culture. Therefore, POC production, as traditionally calculated from quota and growth rate, is a meaningful parameter. A constant quota has been regularly assumed but not tested. This study is one of the few exceptions, and it has strengthened the justification for the usage of POC quota collected at the final day of the experiment. The data (growth rate and PIC and POC production rates) and the treatment at an intermediate CO_2_ level (444 μatm) in addition to those by Bianco et al. (2025) have allowed for a better characterization of the response by *H. carteri* to increasing CO_2_. Growth rate and POC production are likely CO_2_ limited at pre‐industrial CO_2_ concentrations (295 μatm) but likely not proton inhibited at 600 μatm. Finally, the comparison of *H. carteri* with other species cultured under different CO_2_ levels suggests that this species is less sensitive to increasing CO_2_ than previously assumed.

## AUTHOR CONTRIBUTIONS

**Stefania Bianco:** Conceptualization (lead); data curation (lead); formal analysis (equal); funding acquisition (supporting); investigation (lead); methodology (equal); project administration (equal); software (equal); supervision (equal); validation (equal); visualization (lead); writing – original draft (lead); writing – review and editing (equal). **Manuela Bordiga:** Conceptualization (lead); data curation (equal); formal analysis (equal); funding acquisition (lead); investigation (lead); methodology (lead); project administration (lead); resources (lead); software (equal); supervision (equal); validation (equal); visualization (lead); writing – original draft (equal); writing – review and editing (equal). **Gerald Langer:** Conceptualization (equal); data curation (equal); formal analysis (lead); funding acquisition (supporting); investigation (lead); methodology (equal); project administration (equal); resources (supporting); supervision (equal); validation (equal); visualization (lead); writing – original draft (equal); writing – review and editing (equal). **Patrizia Ziveri:** Funding acquisition (supporting); resources (supporting); writing – review and editing (equal). **Federica Cerino:** Formal analysis (supporting); methodology (supporting); resources (supporting); supervision (supporting); writing – review and editing (equal). **Vincenzo Alessandro Laudicella:** Data curation (supporting); formal analysis (supporting); methodology (supporting); resources (supporting); writing – original draft (supporting); writing – review and editing (equal). **Federica Relitti:** Data curation (supporting); formal analysis (supporting); methodology (supporting); resources (supporting); writing – original draft (supporting); writing – review and editing (equal). **Andrea Di Giulio:** Funding acquisition (equal); writing – review and editing (equal). **Claudia Lupi:** Conceptualization (equal); funding acquisition (equal); resources (supporting); supervision (supporting); writing – review and editing (equal).

## FUNDING INFORMATION

This work was funded by MUR for ECORD‐IODP Italia 2018 awarded to Manuela Bordiga with the project “Geochemistry and marine biology united to refine climate models” conducted at the National Institute of Oceanography and Applied Geophysics (OGS) and by the Italian national inter‐university PhD course in Sustainable Development and Climate change (link: www.phd‐sdc.it) awarded to Stefania Bianco. The project was also supported by Claudia Lupi and Andrea Di Giulio with University of Pavia Research Funds (FAR 2021–2023) and by the Okada‐McIntyre Graduate Research Fellowship of INA awarded to Stefania Bianco. Gerald Langer acknowledges funding from the Spanish Ministry of Universities through a Maria Zambrano grant and the Generalitat de Catalunya (MERS, 2021 SGR 00640). This work contributes to the ICTA‐UAB “María de Maeztu” Programme for Units of Excellence of the Spanish Ministry of Science and Innovation (CEX2019‐000940‐M).

This paper and related research have been conducted during and with the support of the Italian national inter‐university PhD course in Sustainable Development and Climate change (link: www.phd‐sdc.it), and it is part of Stefania Bianco's Ph.D. thesis.

## Supporting information


**Table S1.** Summary of all the parameters analyzed in this study and in Bianco et al. (2025), along with the start dates of each experiment conducted in triplicate in the PBRs.
**Table S2.** Data set derived from the three experiments. The reported values for total alkalinity (TA) and dissolved inorganic carbon (DIC) are the averages of six measurements, two for each replicate. *SD*, standard deviation. *From Bianco et al. (2025).
**Table S3.** Summary of *Helicosphaera carteri* coccosphere and protoplast geometry data obtained from the macro in ImageJ Software used in this work. The values reported for each parameter are the averages of three replicates. *SD*, standard deviation; ∅, coccosphere average size; ⊝, protoplast average size.

## Data Availability

The Java script used here within ImageJ software is available on GitHub: https://github.com/mbordiga/Coccoliths (last access: 27 August 2024; https://doi.org/10.5281/zenodo.15122548, mbordiga, 2025). All datasets containing morphometric measurements will be available upon reasonable request.
