# Supplementary material for: Helicosphaera carteri (Prymnesiophyceae) under high carbon dioxide: An experimental study
Source: J Phycol. 2025 Nov 24;61(6):1576–90. doi: 10.1111/jpy.70103 (PMC12718441; doi:10.1111/jpy.70103)
Supplement: Supplementary file 1 — Table S1. Summary of all the parameters analyzed in this study and in Bianco et al. (2025), along with the start dates of each experiment conducted in triplicate in the PBRs. Table S2. Data set derived from the three experiments. The reported values for total alkalinity (TA) and dissolved inorganic carbon (DIC) are the averages of six measurements, two for each replicate. SD, standard deviation. *From Bianco et al. (2025). Table S3. Summary of Helicosphaera carteri coccosphere and protoplast geometry data obtained from the macro in ImageJ Software used in this work. The values reported for each parameter are the averages of three replicates. SD, standard deviation; ∅, coccosphere average size; ⊝, protoplast average size. [file JPY-61-1576-s001.docx]

**Supporting information for:**

***Helicosphaera carteri* (Prymnesiophyceae) under high carbon dioxide: an experimental study^(1)^**

Bianco S.^†1^, Bordiga M.^†2^, Langer G.^(2),3^, Ziveri P.^3,4^, Cerino F.^2^, Relitti F.^2^, Laudicella V. A.^2^, Di Giulio A.^1^, Lupi C.^1^

^1^ Department of Earth and Environmental Sciences, University of Pavia, Pavia, 27100, Italy

^2^ National Institute of Oceanography and Applied Geophysics - OGS, Trieste, Italy

^3^ Institute of Environmental Science and Technology (ICTA), Universitat Autònoma de Barcelona (UAB), 08193 Bellaterra, Spain

^4^ Catalan Institution for Research and Advanced Studies (ICREA), 08010 Barcelona, Spain

^†^These authors equally contributed to this work

^(2)^Corresponding author: Gerald Langer gerald.langer@cantab.net – +4915126298311

**Contents of this file**

This supporting information contains:

- **Table S1.** Summary of all the parameters analyzed in this study and in Bianco et al. (2025), along with the start dates of each experiment conducted in triplicate in the PBRs
- **Table S2.** Data set derived from the three experiments. The reported values for total alkalinity (TA) and dissolved inorganic carbon (DIC) are the averages of six measurements, two for each replicate. *SD* = standard deviation. *From Bianco et al. (2025).
- **Table S3.** Summary of *Helicosphaera carteri* coccosphere and protoplast geometry data obtained from the macro in ImageJ Software used in this work. The values reported for each parameter are the averages of three replicates. *SD* = standard deviation; ∅: coccosphere average size; ⊝: protoplast average size.

**Table S1.** Summary of all the parameters analyzed in this study and in Bianco et al. (2025), along with the start dates of each experiment conducted in triplicate in the PBRs.

| **Experiment (µatm of CO_2_)** | **Data** | **Sampling days** | **Reference** | **Starting and ending dates of experiment** |
| --- | --- | --- | --- | --- |
| 295 | Growth rate | *t*_0_-*t_final_* | This work | From 02 to 09 September 2021 |
|  | Coccosphere size | *t*_0_-*t*_6_ | This work |  |
|  |  | *t*_final_ | Bianco et al. (2025) |  |
|  | Coccosphere roundness | *t*_0_-*t*_6_ | This work |  |
|  |  | *t*_final_ | Bianco et al. (2025) |  |
|  | Protoplast size | *t*_0_-*t*_6_ | This work |  |
|  |  | *t*_final_ | Bianco et al. (2025) |  |
|  | Protoplast roundness | *t*_0_-*t*_6_ | This work |  |
|  |  | *t*_final_ | Bianco et al. (2025) |  |
|  | PIC quota | *t*_final_ | Bianco et al. (2025) |  |
|  | POC quota | *t*_0_-*t*_6_ | This work |  |
|  |  | *t*_final_ | Bianco et al. (2025) |  |
|  | PIC and POC production | *t*_final_ |  |  |
|  | PIC:POC | *t*_final_ |  |  |
|  | | | |  |
| 444 | Growth rate | *t*_0_-*t*_final_ | This work | From 22 to 28 July 2021 |
|  | Coccosphere size |  |  |  |
|  | Coccosphere roundness |  |  |  |
|  | Protoplast size |  |  |  |
|  | Protoplast roundness |  |  |  |
|  | POC quota |  |  |  |
|  | POC production | *t*_final_ |  |  |
|  | | | |  |
| 600 | Growth rate | *t*_0_-i_final_ | This work | From 23 to 29 June 2021 |
|  | Coccosphere size | *t*_0_-*t*_6_ | This work |  |
|  |  | *t*_final_ | Bianco et al. (2025) |  |
|  | Coccosphere roundness | *t*_0_-*t*_6_ | This work |  |
|  |  | *t*_final_ | Bianco et al. (2025) |  |
|  | Protoplast size | *t*_0_-i_6_ | This work |  |
|  |  | *t*_final_ | Bianco et al. (2025) |  |
|  | Protoplast roundness | *t*_0_-*t*_6_ | This work |  |
|  |  | *t*_final_ | Bianco et al. (2025) |  |
|  | PIC quota | *t*_final_ | Bianco et al. (2025) |  |
|  | POC quota | i_0_-*t*_6_ | This work |  |
|  |  | *t*_final_ | Bianco et al. (2025) |  |
|  | PIC and POC production | *t*_final_ | This work |  |
|  | PIC:POC | *t*_final_ | Bianco et al. (2025) |  |

**Table S2.** Data set derived from the three experiments. The reported values for total alkalinity (TA) and dissolved inorganic carbon (DIC) are the averages of six measurements, two for each replicate. *SD* = standard deviation. *From Bianco et al. (2025).

| **Exp.** | **CO_2_ level (µatm)** | **TA**  **(µmol ⋅ kg^−¹^)** | **pH NBS** | **CO_2_**  **(µmol ⋅ kg^−^¹)** | **HCO_3_^-^ (µmol ⋅ kg^−^¹)** | **CO_3_^2-^**  **(µmol ⋅ kg^−^¹)** | **DIC**  **(µmol ⋅ kg^−^¹)** | **Ω calcite** |
| --- | --- | --- | --- | --- | --- | --- | --- | --- |
| 1* | 294.56 | 1853.82 | 8.18 | 9.8 | 1413.49 | 141.44 | 1677.5 | 3.38 |
| *SD* | 17.84 | 166.93 | 0.03 | 121.85 | 106.02 | 16.62 | 140.85 | 0.40 |
| 2 | 444.48 | 1762.30 | 8.02 | 14.7 | 1441.83 | 97.58 | 1621.2 | 2.33 |
| *SD* | 84.45 | 215.26 | 0.03 | 198.52 | 188.96 | 6.79 | 197.05 | 0.16 |
| 3* | 601.5 | 1452.54 | 7.81 | 19.9 | 1213.70 | 51.72 | 1374.7 | 1.24 |
| *SD* | 59.74 | 146.41 | 0.06 | 156.94 | 144.50 | 13.38 | 142.06 | 0.32 |

**Table S3.** Summary of *Helicosphaera carteri* coccosphere and protoplast geometry data obtained from the macro in ImageJ Software used in this work. The values reported for each parameter are the averages of three replicates. *SD* = standard deviation; ∅: coccosphere average size; ⊝: protoplast average size.

| **CO_2_**  **(µatm)** | **Day**  **(t)** | **∅**  **(µm)** | **⊝**  **(µm)** | **RD_coccosphere_** | **RD_protoplast_** | **POC_geometry_**  **(pg ⋅ cell^−1^)** | **No. of analyzed specimens** |
| --- | --- | --- | --- | --- | --- | --- | --- |
| 295 | 0 | 17.95 | 11.52 | 0.86 | 0.86 | 108.22 | 304 |
|  | *SD* | 0.19 | 0.73 | 0.01 | 0.03 | 10.28 |  |
|  | 1 | 17.75 | 11.25 | 0.84 | 0.88 | 101.48 | 324 |
|  | *SD* | 0.38 | 0.30 | 0.05 | 0.01 | 7.97 |  |
|  | 2 | 18.18 | 11.49 | 0.88 | 0.88 | 107.76 | 316 |
|  | *SD* | 0.34 | 0.28 | 0.01 | 0.02 | 8.13 |  |
|  | 3 | 18.14 | 11.43 | 0.88 | 0.89 | 106.97 | 316 |
|  | *SD* | 0.19 | 0.20 | 0.01 | 0.02 | 5.58 |  |
|  | 4 | 18.39 | 11.36 | 0.89 | 0.90 | 105.35 | 340 |
|  | *SD* | 0.16 | 0.06 | 0.01 | 0.02 | 3.09 |  |
|  | 5 | 18.37 | 11.35 | 0.89 | 0.88 | 104.43 | 313 |
|  | *SD* | 0.17 | 0.08 | 0.01 | 0.03 | 2.70 |  |
|  | 6 | 18.10 | 11.60 | 0.88 | 0.90 | 111.62 | 316 |
|  | *SD* | 0.78 | 0.22 | 0.04 | 0.03 | 7.69 |  |
|  | 7 | 18.10 | 11.45 | 0.89 | 0.90 | 108.14 | 312 |
|  | *SD* | 0.25 | 0.19 | 0.02 | 0.03 | 5.42 |  |
| 444 | 0 | 17.88 | 11.66 | 0.89 | 0.91 | 11.64 | 330 |
|  | *SD* | 0.18 | 0.25 | 0.01 | 0.01 | 7.46 |  |
|  | 1 | 18.35 | 11.80 | 0.88 | 0.90 | 118.27 | 322 |
|  | *SD* | 0.85 | 0.37 | 0.01 | 0.01 | 9.90 |  |
|  | 2 | 18.05 | 11.60 | 0.86 | 0.88 | 111.26 | 309 |
|  | *SD* | 0.35 | 0.10 | 0.04 | 0.04 | 4.20 |  |
|  | 3 | 18.35 | 11.53 | 0.88 | 0.88 | 109.01 | 316 |
|  | *SD* | 0.23 | 0.12 | 0.01 | 0.02 | 2.85 |  |
|  | 4 | 18.32 | 11.44 | 0.87 | 0.90 | 108.16 | 316 |
|  | *SD* | 0.18 | 0.20 | 0.02 | 0.02 | 3.70 |  |
|  | 5 | 18.17 | 11.80 | 0.86 | 0.89 | 116.79 | 315 |
|  | *SD* | 0.34 | 0.38 | 0.01 | 0.03 | 9.45 |  |
|  | 6 | 18.16 | 11.61 | 0.87 | 0.90 | 112.56 | 212 |
|  | SD | 0.20 | 0.38 | 0.01 | 0.02 | 9.01 |  |
| 600 | 0 | 18.70 | 12.42 | 0.89 | 0.94 | 136.62 | 314 |
|  | *SD* | 0.69 | 0.25 | 0 | 0.15 | 16.45 |  |
|  | 1 | 17.88 | 12.10 | 0.88 | 0.97 | 128.27 | 319 |
|  | *SD* | 0.30 | 0.42 | 0.02 | 0.15 | 13.42 |  |
|  | 2 | 17.81 | 12.27 | 0.87 | 0.97 | 133.64 | 318 |
|  | *SD* | 0.17 | 0.41 | 0.01 | 0.15 | 13.96 |  |
|  | 3 | 17.90 | 12.10 | 0.88 | 0.96 | 128.71 | 321 |
|  | *SD* | 0.34 | 0.52 | 0.01 | 0.14 | 14.30 |  |
|  | 4 | 17.83 | 11.94 | 0.88 | 0.95 | 122.47 | 321 |
|  | *SD* | 0.03 | 0.66 | 0,01 | 0.14 | 13.05 |  |
|  | 5 | 18.30 | 12.32 | 0.89 | 0.97 | 136.07 | 320 |
|  | *SD* | 0.22 | 0.52 | 0.01 | 0.14 | 18.11 |  |
|  | 6 | 17.92 | 11.81 | 0.88 | 0.91 | 118.51 | 312 |
|  | *SD* | 0.66 | 0.27 | 0.005 | 0.014 | 6.41 |  |

**References**

Bianco, S., Bordiga, M., Langer, G., Ziveri, P., Cerino, F., Di Giulio, A. S., & Lupi, C. (2025). Low sensitivity of a heavily calcified coccolithophore under increasing CO_2_: The case study of *Helicosphaera carteri. Biogeosciences*, *22,* 1821–1837*.* <https://doi.org/10.5194/bg-22-1821-2025>
